# Molecular Mechanisms of APOL1-Associated Kidney Disease

**DOI:** 10.3390/ijms27062863

**Published:** 2026-03-21

**Authors:** Charlotte Delrue, Reinhart Speeckaert, Marijn M. Speeckaert

**Affiliations:** 1 Department of Nephrology, Ghent University Hospital, 9000 Ghent, Belgium; charlotte.delrue@ugent.be; 2 Department of Dermatology, Ghent University Hospital, 9000 Ghent, Belgium; reinhart.speeckaert@ugent.be; 3 Research Foundation-Flanders (FWO), 1000 Brussels, Belgium

**Keywords:** APOL1, chronic kidney disease, podocytes, interferon signaling, mitochondrial dysfunction, proteostasis, precision nephrology, gene–environment interaction

## Abstract

The discovery of apolipoprotein L1 (APOL1) risk polymorphisms has significantly changed our knowledge of kidney disease susceptibility and development in African American populations. Several non-diabetic kidney disorders, such as focal segmental glomerulosclerosis (FSGS), collapsing glomerulopathy, HIV-associated nephropathy (HIVAN), and accelerated chronic kidney disease (CKD) development, are significantly more likely to occur in people with two coding variations, G1 and G2. The significance of context-dependent pathogenic processes is highlighted by the poor penetrance and remarkable phenotypic variety of APOL1-associated kidney disease, despite its substantial impact. This review synthesizes current knowledge of APOL1 biology through a molecular framework, emphasizing gain-of-toxic-function effects of risk variants in podocytes, dysregulated ion fluxes, mitochondrial dysfunction, impaired proteostasis, and activation of innate immune and inflammatory signaling pathways. We describe how the well-recognized “second-hit” paradigm has a biological basis, driven by strong inducibility by interferons and immunological activation, as well as strict basal regulation of APOL1 expression. Lastly, we explore future approaches to precision nephrology and highlight translational advancements, such as *APOL1* gene-silencing techniques. This review provides a mechanistic roadmap for translating APOL1 biology into targeted therapeutic strategies by integrating genetics, cell biology, immunology, and systems-level approaches.

## 1. Introduction

The identification of risk variants in the apolipoprotein L1 (APOL1) gene is one of the greatest advances in the genetic understanding of kidney disease. Genome-wide association studies (GWASs) in populations of African ancestry first demonstrated that two coding variants in the APOL1 gene, G1 and G2, are strongly associated with an increased risk of non-diabetic kidney disease (DKD), including focal segmental glomerulosclerosis (FSGS), HIV-associated nephropathy (HIVAN), collapsing glomerulopathy, and accelerated chronic kidney disease (CKD) progression [1,2,3,4]. Individuals carrying two APOL1 risk alleles exhibit a several-fold increased lifetime risk of kidney disease compared with those carrying zero or one risk allele [2,3]. Despite the strong effect size of APOL1 high-risk genotypes, disease penetrance is incomplete: the majority of individuals with two risk alleles do not develop overt kidney disease [5].

Mechanistic studies in cellular and transgenic animal models, including podocyte-specific APOL1 G1/G2 expression models that recapitulate proteinuria and glomerulosclerosis, demonstrate that APOL1 risk variants exert gain-of-toxic-function effects, particularly in podocytes [6,7]. These effects include dysregulated ion fluxes, mitochondrial depolarization, cytoskeletal disruption, impaired autophagy, and activation of inflammatory and stress-response pathways [8]. Emerging evidence suggests that endothelial and tubular epithelial cells may also contribute to disease pathogenesis, reinforcing the concept of APOL1-associated kidney disease as a multicellular disorder driven by shared molecular stress programs [9].

From a translational standpoint, APOL1-associated kidney disease represents a prototype for precision nephrology. The strong causal link between a defined genetic variant and kidney injury has enabled the development of targeted therapeutic strategies, including antisense oligonucleotides and RNA interference approaches designed to reduce APOL1 expression [6,10]. These therapies, now advancing through clinical development, exemplify a shift toward genotype-informed, mechanism-based treatment of kidney disease.

Therefore, this review aims to provide a comprehensive mechanistic overview of APOL1-associated kidney disease by integrating evidence from clinical observations, experimental cell-based systems, and transgenic animal models. Particular emphasis is placed on the molecular pathways through which APOL1 risk variants induce injury in renal cells. Experimental systems, including immortalized human podocyte cell lines, induced pluripotent stem cell-derived kidney organoids, and transgenic mouse models expressing human APOL1 risk variants, have been instrumental in elucidating the molecular mechanisms of APOL1-mediated kidney injury [6,9,10]. By integrating experimental findings with human genetic and clinical studies, this review outlines how dysregulated ion transport, mitochondrial dysfunction, impaired proteostasis, and activation of innate immune signaling collectively contribute to podocyte injury and to progressive glomerulonephritis. Besides that, animal models in experiments have been indispensable for outlining the concept that APOL1 risk alleles confer a gain-of-toxic function and for revealing that expression of APOL1 G1 or G2 alleles specifically in podocytes can lead to proteinuria and glomerulosclerosis. Hence, integrating mechanistic insights from experiments with clinical observations is essential not only for understanding the pathophysiology of APOL1-associated kidney disease but also for guiding the development of new targeted treatment options.2. Clinical Spectrum and Genetic Architecture of APOL1-Associated Kidney Disease

### 1.1. APOL1 G1 and G2 Variants and Population Distribution

The genetic architecture of APOL1-associated kidney disease is primarily driven by two coding risk variants, G1 and G2, which originated and expanded in populations of recent African ancestry under intense selective pressure. The G1 risk allele consists of two missense substitutions in near-complete linkage disequilibrium (S342G and I384M), whereas the G2 allele is defined by a two-amino acid deletion (N388/Y389). Both variants alter the C-terminal domain of the APOL1 protein, a region critical for its interaction with trypanosomal serum resistance-associated proteins and for intracellular cytotoxicity [1,2,11]. APOL1 risk alleles are largely absent in European, East Asian, and Indigenous American populations, but are common in West and sub-Saharan African populations, with allele frequencies approaching 30–40% in some regions [1,12]. Approximately 13–15% of African Americans carry two APOL1 risk alleles (high-risk genotype), placing them at substantially increased risk for kidney disease [3]. The strong ancestry specificity of APOL1-associated risk reflects historical exposure to Trypanosoma brucei infection and represents one of the clearest examples of recent positive selection shaping modern disease susceptibility [11,13]. Importantly, APOL1-associated kidney disease follows a recessive genetic model, with increased disease risk largely confined to individuals carrying two risk alleles (G1/G1, G1/G2, or G2/G2). Heterozygous carriers generally do not exhibit an increased risk of kidney disease, underscoring the necessity of high-risk genotypes for pathogenicity [2,3].

### 1.2. Clinical Phenotypes Associated with APOL1 High-Risk Genotypes

At the population level, APOL1 risk variants are associated with faster CKD progression and increased risk of kidney failure, even in the absence of a specific glomerular diagnosis [3,5]. Many individuals are clinically labeled as having hypertension-attributed CKD, although accumulating evidence suggests that APOL1-associated podocyte injury may be the primary driver of disease in a substantial subset of these patients [3,14]. APOL1 high-risk genotypes predispose to a spectrum of kidney diseases rather than a single clinicopathologic entity. The strongest and most reproducible associations have been observed with FSGS, HIVAN, collapsing glomerulopathy, and progressive CKD [2,15,16]. The major APOL1-associated clinical phenotypes and their triggering contexts are summarized in Table 1.

Among these, FSGS represents the most extensively studied phenotype, and recent reviews have summarized how APOL1 risk variants contribute to podocyte injury, glomerulosclerosis, and clinical heterogeneity in this disease [14,21]. APOL1 high-risk genotypes are highly prevalent in patients with primary FSGS and secondary forms of FSGS not attributable to adaptive or genetic causes. Histologically, APOL1-associated FSGS often displays collapsing features, marked podocyte injury, and rapid progression to kidney failure [4,22]. Notably, APOL1 risk variants account for a substantial proportion of cases previously labeled as “idiopathic” FSGS in individuals of African ancestry. Moreover, in the absence of antiretroviral therapy, HIV-positive individuals with two APOL1 risk alleles have an extraordinarily high risk of developing HIVAN, with odds ratios exceeding 20–30 in some studies [16,23]. HIVAN is characterized by a collapsing glomerulopathy, tubular microcystic dilation, and severe proteinuria, and its strong association with APOL1 genotype provides early evidence supporting a gene–environment interaction model. Beyond HIVAN, APOL1 high-risk genotypes have been implicated in collapsing glomerulopathy occurring in diverse inflammatory contexts, including viral infections (e.g., SARS-CoV-2), IFN exposure, autoimmune disease, and malignancy-associated immune activation [22,24,25]. These observations reinforce the notion that APOL1-associated kidney disease reflects a shared molecular vulnerability that can be unmasked by heterogeneous clinical insults.

### 1.3. Incomplete Penetrance and the “Second-Hit” Concept

Longitudinal cohort studies demonstrate that most individuals carrying two APOL1 risk alleles do not develop clinically overt kidney disease during their lifetimes [5]. This observation has led to the widely accepted “second-hit” hypothesis, which posits that additional genetic, environmental, or inflammatory factors are required to trigger disease onset in genetically susceptible individuals. Several lines of evidence support this model. Experimental studies demonstrate that APOL1 risk variant expression alone may be insufficient to cause kidney injury unless expression levels exceed a pathogenic threshold, further supporting a dose- and context-dependent model of toxicity [6]. Genetic and epigenetic variation are increasingly recognized as factors in the heterogeneity of symptoms within the same disease. They can affect, among others, transcriptional regulation, cellular stress responses, and even injury resistance [19,26,27]. Several factors that can modify have been identified, though some lack sufficient evidence to make them strong. For example, by analyzing integrative genomics, the NEPTUNE group discovered that regulatory variants in inflammatory and interferon pathways could alter APOL1 expression and associated transcriptional pathways, indicating that immune activation could affect genetic risk [19]. Besides that, other research has proposed that genes responsible for mitochondrial function and cellular stress responses might be the ones that modify APOL1, which is associated with metabolic injury. These proposals are consistent with experimental evidence that APOL1 risk variants are associated with mitochondrial dysfunction and energy stress in podocytes [9,20]. Epigenetic changes are also likely to be involved in modulating disease phenotypes. For example, altered DNA methylation patterns and chromatin accessibility at immune and metabolic regulatory loci have been found in chronic kidney disease. These, in turn, may influence APOL1 transcriptional responsiveness during inflammation [27]. Nevertheless, lots of these suggested modifiers are still at the nascent stage, and findings have not yet been consistently replicated across independent cohorts. This means there is a clear need for large-scale, multi-ancestry genomic and epigenomic studies to identify which of these modifiers can produce reproducible effects on the susceptibility and progression of APOL1-associated kidney disease. The second-hit paradigm has important clinical implications. It explains the variable age of onset, phenotypic diversity, and unpredictable disease course observed among APOL1 high-risk individuals. Moreover, it highlights opportunities for intervention at multiple levels, including modulating inflammatory triggers, targeting APOL1 expression directly, and identifying protective modifiers that mitigate disease risk Other than the well-known interferon, enriched environments in HIVAN and in collapsing glomerulopathy, there is a possibility that more chronic and low-grade external stressors also play a role in APOL1-associated CKD. This is especially true for patients with progressive non-collapsing disease, often referred to as hypertension, attributed to nephropathy. In these patients, the relevant “second hit” might not be a single, intense inflammatory event but rather a continuous, low-level oxidative and inflammatory stress. Some of the potential factors are obesity, insulin resistance, high salt or Western diet, smoking, chronic vascular stress due to hypertension, and recurrent subclinical inflammatory exposures [14,28,29]. While direct causal evidence remains scarce, these factors remain biologically plausible because they can induce oxidative stress, endothelial dysfunction, mitochondrial injury, and inflammatory signaling, thereby lowering the threshold at which APOL1 risk-variant expression becomes pathogenic [9,30,31]. According to this model, kidney disease related to APOL1 may develop due to a sudden injury caused by interferon, which is only a short-term factor, but could also be a consequence of the cumulative effects of chronic metabolic and inflammatory factors that will gradually increase the vulnerability of podocytes, endothelial, and tubular cells [28,31]. This option is of great significance for further scientific studies since it supports the idea that prevention of diseases might require the combination of genetic approaches with the implementation at the level of the whole population of methods limiting diet, improving metabolic health, optimizing blood pressure, controlling drug intake, and other factors that contribute to inflammation and oxidative stress throughout life [14,29].

Experimental models provide strong mechanistic support for the biological plausibility of the ‘second-hit’ paradigm. In transgenic mouse models expressing APOL1 risk alleles in podocytes, renal injury is markedly exacerbated when animals are exposed to inflammatory stimuli that activate interferon signaling. In experimental systems, for example, interferon treatment and viral infection significantly increase APOL1 levels and accelerate glomerulonephritis [6,7]. Similarly, in vitro studies using cultured podocytes demonstrate that exposure to inflammatory cytokines or interferons enhances APOL1-mediated cytotoxicity and increases APOL1 expression to pathogenic levels [10,32]. These findings provide experimental support for the gene–environment interaction model suggested by human studies and show that context is a major factor in APOL1 toxicity.

## 2. Molecular Mechanisms of APOL1-Mediated Kidney Injury

A large body of experimental evidence derived from cell culture systems, genetically engineered mouse models, and human kidney transcriptomic studies demonstrates that APOL1 risk variants initiate multiple interconnected cellular stress pathways that ultimately lead to podocyte injury and glomerular dysfunction [6,8,10]. Although individual mechanisms have frequently been investigated in isolation, current evidence indicates that APOL1 toxicity arises from a complex network of interconnected cellular stress pathways. The upcoming paragraphs outline the main molecular pathways by which APOL1 risk variants trigger cellular injury and the progression of kidney disease.

### 2.1. APOL1 Expression, Regulation, and Cellular Localization

A defining feature of APOL1-associated kidney disease is the stringent regulation of APOL1 expression under physiological conditions coupled with its marked inducibility in inflammatory states. In healthy individuals, basal APOL1 expression within the kidney is low, particularly in glomerular cells, with transcript and protein levels often near the limits of detection [1,2,15]. APOL1 is a highly interferon-responsive gene, and its transcription is robustly induced by both type I (IFN-α/β) and type II (IFN-γ) interferons through activation of the Janus kinase and signal transducer and activator of transcription (JAK–STAT) signaling pathway [7,26,33]. Both the magnitude and duration of APOL1 induction appear to be critical, supporting a threshold-dependent model in which transient or modest upregulation may be tolerated, whereas sustained high-level expression precipitates cellular injury [5,6].

Although APOL1 was initially characterized as a circulating innate immune protein synthesized primarily by the liver, it is now well established that APOL1 is also expressed locally within the kidney in a cell-type-specific manner [13]. Among renal cell populations, podocytes show the strongest evidence for pathogenic relevance. Single-cell RNA sequencing studies of human kidney tissue reveal enrichment of APOL1 transcripts in podocytes, particularly in inflammatory and disease states. Podocyte-specific expression of APOL1 risk variants is sufficient to induce proteinuria, foot process effacement, and progressive glomerulosclerosis in experimental models, firmly establishing podocytes as the principal effector cell in APOL1-associated kidney disease [34,35,36]. In addition to podocytes, glomerular endothelial cells express APOL1, especially following interferon stimulation [7]. Endothelial APOL1 expression has been linked to microvascular dysfunction, altered barrier integrity, and maladaptive crosstalk with podocytes, suggesting that APOL1-mediated injury is not strictly podocyte autonomous [28]. Tubular epithelial cells, particularly proximal tubule cells, exhibit relatively low basal APOL1 expression but demonstrate inducible expression under inflammatory, ischemic, or toxic stress [7]. While tubular expression alone is unlikely to initiate disease, it may contribute to tubulointerstitial injury, maladaptive repair, and fibrosis during disease progression, especially in advanced CKD [37]. These findings support a multicellular model in which podocytes represent the primary site of injury, with endothelial and tubular compartments modulating disease severity and chronicity. A major issue still unresolved in the field is how much circulating versus locally expressed APOL1 contributes to kidney injury [18]. APOL1 is mainly produced by the liver and released into the bloodstream attached to high-density lipoprotein (HDL) particles and trypanolytic complexes, which are part of the innate immune system response to Trypanosoma species [17]. However, there is growing experimental evidence that circulating APOL1 alone does not cause kidney injury. Podocytes from selective transgenic mouse models expressing APOL1 risk variants produce proteinuria and glomerulosclerosis even in the absence of circulating human APOL1, thereby supporting a cell-autonomous mechanism of toxicity [6,38]. Additionally, transcriptomic and single-cell analyses of human kidney tissues have shown that APOL1 expression can be induced across different renal cell types under inflammatory conditions [7,39]. This tells us that APOL1 production locally within renal cells, especially podocytes, is the key driver of toxic effects. Nevertheless, the possible role of circulating APOL1 in disease susceptibility remains under investigation.

The subcellular localization of APOL1 further shapes its pathogenic potential and is highly dynamic, depending on expression level and cellular context. APOL1 lacks a classical signal peptide and localizes predominantly to intracellular membranes under basal conditions, particularly the endoplasmic reticulum, where it is thought to participate in membrane and lipid homeostasis [13,17,26]. Upon interferon-induced upregulation or overexpression, APOL1 (especially the G1 and G2 risk variants) redistributes to additional compartments, including mitochondria, endolysosomal membranes, and the plasma membrane [7]. Mitochondrial localization of APOL1 risk variants has been associated with mitochondrial depolarization, impaired oxidative phosphorylation, and increased reactive oxygen species (ROS) generation, linking altered localization to metabolic stress and energetic failure [9]. At the plasma membrane and within endolysosomal compartments, APOL1 functions as a pH-sensitive cation channel that promotes potassium efflux, disrupts ionic homeostasis, and activates downstream inflammatory and lytic cell death pathways [10,40].

Regulation of APOL1 extends beyond interferon-driven transcription. Epigenomic studies indicate that chromatin accessibility and histone modification patterns at the APOL1 locus vary by cell type and inflammatory state, influencing both basal expression and inducibility [34,35]. Post-transcriptional mechanisms, including regulation of mRNA stability by RNA-binding proteins and microRNAs, further modulate APOL1 abundance, although these pathways remain incompletely defined [28]. At the protein level, APOL1 undergoes rapid turnover under basal conditions, whereas risk variants appear to exhibit altered proteostasis, including impaired degradation and increased susceptibility to misfolding under cellular stress. Notably, G1 and G2 variants do not consistently differ from the reference allele in expression level, indicating that pathogenicity arises primarily from qualitative differences in protein behavior rather than increased abundance per se [6,26]. While podocytes are the dominant effector cell type, the relative contributions of endothelial and tubular compartments likely vary by disease stage and inflammatory context. Because APOL1 expression occurs across multiple renal cell types, the current evidence supporting compartment-specific contributions to APOL1-mediated kidney injury is summarized in Table 2.

### 2.2. Gain-of-Toxic-Function Paradigm of APOL1 Risk Variants

Accumulating genetic, experimental, and clinical evidence strongly supports a gain-of-toxic-function model for APOL1-associated kidney disease, rather than a loss-of-function model. Several lines of evidence argue against a loss-of-function mechanism. First, individuals completely lacking APOL1 expression due to null variants do not exhibit an increased risk of kidney disease, indicating that APOL1 is not essential for normal renal homeostasis. Second, APOL1 risk variants do not lead to reduced expression or impaired secretion of the protein under basal conditions. Instead, they retain trypanolytic activity and normal circulating function. Third, experimental deletion of APOL1 in model systems fails to reproduce the kidney disease phenotypes seen with expression of the risk variants [1,28].

Direct experimental evidence for gain-of-toxic-function comes from transgenic and cell-based models demonstrating that expression of APOL1 risk variants is sufficient to induce kidney injury, whereas expression of the reference allele is largely benign at comparable levels. Podocyte-specific transgenic expression of human APOL1 G1 or G2 variants results in proteinuria, podocyte foot process effacement, collapsing glomerulopathy, and progressive glomerulosclerosis, while expression of the reference allele does not produce similar pathology [6]. Importantly, these disease phenotypes occur despite similar expression levels of reference and risk alleles, underscoring that qualitative differences in protein behavior, rather than increased abundance, drive toxicity. Complementary in vitro studies further demonstrate that APOL1 risk variants induce cellular injury through mechanisms including dysregulated ion fluxes, mitochondrial dysfunction, endoplasmic reticulum (ER) stress, and inflammatory cell death pathways, all of which are consistent with a toxic gain-of-function [40].

Although G1 and G2 variants differ structurally, both pose similar risks of kidney disease at the population level and exhibit broadly overlapping toxic phenotypes at the cellular level. Experimental studies suggest that G1 and G2 share common downstream injury pathways, including potassium efflux, mitochondrial depolarization, and inflammasome activation, supporting a convergent mechanism of toxicity [40]. Nonetheless, subtle differences between G1 and G2 have been reported. For example, some studies suggest variant-specific effects on protein stability, subcellular localization, or magnitude of cytotoxicity under specific experimental conditions, although these differences have not translated into clearly distinct clinical phenotypes [26,28].

A characteristic feature of APOL1 risk variant toxicity is its dose dependence and strong context dependence. Expression of APOL1 risk variants at low levels is often tolerated, whereas higher expression levels precipitate cellular injury and death. This dose-dependent effect has been shown in multiple model systems, including inducible expression in podocytes and renal epithelial cells, with toxicity occurring only when expression exceeds a critical threshold [6]. This threshold-dependent toxicity explains the incomplete penetrance of APOL1-associated kidney disease [4,15,24]. The major molecular mechanisms by which APOL1 risk variants injure renal cells are summarized in Table 3 and discussed below.

### 2.3. Ion Channel Activity and Membrane Permeabilization

One of the most widely discussed mechanistic models in the current literature proposes that APOL1 acts as a pH-dependent, active cation channel. Studies using human cultured epithelial cells expressing APOL1 risk variants (G1 or G2) have shown that these variants can localize to the plasma membrane, where they form active ion channels. Channel activity at the cell surface disrupts intracellular ionic homeostasis, including potassium efflux, and has been associated with cytotoxicity in cellular models, suggesting that plasma membrane APOL1 may represent an early event that contributes to downstream injury pathways or enhances the cellular effects of channel activity in mammalian cells. Initial biophysical reconstitution experiments gave crucial evidence for this model. These studies demonstrated that recombinant APOL1 can insert into artificial lipid bilayers in a pH-dependent manner and produce cation-selective conductance. The results are consistent with a two-step mechanism in which low pH conditions facilitate the membrane insertion of APOL1, whereas subsequent neutralization opens the channel and significantly increases ion conductance [40]. One of the earliest cellular consequences of APOL1 risk-variant expression is disruption of intracellular potassium homeostasis. G1 and G2 expression produce a marked net efflux of intracellular K^+^, and experimental manipulation of extracellular potassium or pathways responsive to K^+^ depletion modulates cytotoxicity. These ionic perturbations are accompanied by prominent cell swelling and plasma membrane injury, which aligns with a membrane-permeabilizing mechanism and provides a mechanistic bridge from channel activity to overt cell death phenotypes [10,43]. Several experimental studies using heterologous expression systems and transgenic podocyte models demonstrate that aberrant K^+^ efflux is among the most reproducible early events in APOL1 toxicity [6,10,40]. This observation suggests that potassium loss may represent a unifying mechanism linking diverse downstream injury pathways across experimental systems [28,29].

Loss of intracellular K^+^ is also a well-established trigger for inflammatory cell death pathways, and the emerging APOL1 literature increasingly links channel-driven ionic imbalance to inflammasome activation and lytic forms of cell death. In APOL1-associated podocytopathy models and human high-risk settings, inflammasome-related signaling has been implicated downstream of APOL1, including activation of pathways converging on caspase-1 and gasdermin-mediated membrane permeabilization (pyroptosis), which provides a mechanistic route from ionic disequilibrium to inflammatory, lytic cell death programs. APOL1 channel activity (or channel-associated membrane permeabilization) promotes K^+^ efflux, thereby lowering intracellular potassium concentration and facilitating inflammasome assembly/activation. Gasdermin pore formation and membrane rupture can then amplify ionic dysregulation and produce rapid cellular collapse [6,44]. Importantly, while the precise sequence and relative contributions of pyroptotic, necrotic, and other death programs likely vary by cell type and experimental context, the recurring observation is that membrane permeabilization and K^+^ loss are proximal events that can drive multiple terminal pathways. These mechanisms are particularly relevant to podocyte vulnerability because podocytes are highly specialized, terminally differentiated cells that rely on tightly regulated membrane potential, cytoskeletal architecture, and slit diaphragm integrity to maintain the glomerular filtration barrier. Podocytes also appear especially susceptible to APOL1 risk-variant injury when APOL1 expression is induced (e.g., by interferon signaling) and when APOL1 reaches or is recruited to membrane compartments where channel activity becomes pathogenic [7,10]. In vivo, podocyte-restricted expression of APOL1 risk variants is sufficient to produce proteinuria and progressive glomerulonephritis, establishing that podocytes can serve as a primary cellular locus in which membrane permeabilization and ionic dysregulation translate into barrier failure [6,8]. Recent work further supports the importance of surface expression and haplotype context in modulating podocyte cytotoxicity, consistent with the idea that the degree of plasma membrane localization (and thus the probability of channel activity at the cell surface) can shape the magnitude of injury [8,40].

### 2.4. Mitochondrial Dysfunction and Metabolic Stress

APOL1 risk variants directly impair mitochondrial structure and function, particularly in metabolically demanding renal cell types. Studies using several models to assess APOL1 G1 and G2 variants demonstrated that these variants can localize to the mitochondria, either directly or indirectly, where they affect mitochondrial membrane stability and disrupt nitrogen homeostasis by promoting oxidative stress and reducing mitochondrial function. Experimental studies in cultured podocytes and APOL1 transgenic mouse models demonstrate that mitochondrial depolarization, ROS production, and impaired oxidative phosphorylation are central mediators of APOL1-induced cytotoxicity [9,20,30].

One of the earliest and most consistently observed mitochondrial abnormalities associated with APOL1 risk variants is mitochondrial membrane depolarization. The emergence of G1 or G2 isoforms in kidney podocytes and epithelial cell types rapidly depletes mitochondrial potential, owing to a loss of membrane integrity and the inability to maintain proton gradients [26]. The depolarization observed with G1 or G2 expression is accompanied by an elevation in ROS production, including superoxide, from within mitochondria and from other cells. These ROS subsequently injure the DNA, proteins, and lipids of the mitochondria. Of particular relevance to the mitochondrial injury caused by APOL1 variants is the idea that the generation of ROS from mitochondrial dysfunction is both a cause and an effect of the injury, thus creating a feed-forward cycle in which oxidative stress continues to worsen the original mitochondrial toxicity and cellular death caused by APOL1 variants [28].

Beyond membrane depolarization, APOL1 risk variants exert profound effects on oxidative phosphorylation (OXPHOS). Studies that have employed the Seahorse flux analysis method and other types of bioenergetic assays have found that both individuals with APOL1 G1 and G2 exhibit lower amounts of both basal and maximal mitochondrial respiration, reduced spare capacity for respiration and a reduced amount of ATP production than would be expected if both were of equal expression levels to the control APOL1 allele [30]. If cells cannot generate enough ATP through OXPHOS, their ability to respond to the increased energetic demands of stress is limited, thereby making them more susceptible to secondary stressors, such as inflammatory activation and metabolic challenges.

As a consequence of defective mitochondrial respiration, the APOL1 risk variant-expressing cells undergo metabolic reprogramming characterized by a shift away from oxidative metabolism toward glycolysis and stress-adaptive metabolic programs. Transcriptomic and metabolomic studies have indicated the activation of pathways associated with the integrated stress response, altered NAD^+^/NADH balance, and dysregulated lipid metabolism in APOL1 high-risk contexts [26,34]. While such metabolic reprogramming may initially represent an adaptive response to mitochondrial injury, sustained reliance on less efficient energy pathways ultimately leads to energetic failure, particularly in terminally differentiated cells with limited metabolic plasticity. In podocytes, which require continuous ATP generation to maintain cytoskeletal dynamics and slit diaphragm integrity, energetic insufficiency can precipitate foot process effacement, detachment, and cell loss [45].

Mitochondrial dysfunction also amplifies other APOL1-mediated injury pathways by promoting oxidative stress and energetic failure [46]. In this integrated stress landscape, mitochondrial injury serves as both a primary toxic effect of APOL1 risk variants and a convergence point through which ion channel activity, proteostatic stress, and inflammatory signaling exacerbate cellular damage.

Mitochondrial and metabolic stress serve as mechanisms for connecting an individual’s APOL1 genotype to the progression of CKD at both the tissue and organism levels. Evidence from population studies indicates that people who possess an APOL1 high-risk genotype tend to lose renal function faster and advance sooner to kidney failure than non-carriers, even when no glomerular disease is identified [3,5]. There is substantial evidence that chronic mitochondrial dysfunction is a significant contributor to maladaptive repair processes, leading to tubular atrophy and interstitial fibrosis, two key features of progressive CKD [31]. In APOL1-associated kidney disease, repeated or sustained episodes of mitochondrial injury triggered by inflammatory “second hits” likely accelerate these processes, contributing to irreversible nephron loss over time.

Factors that characterize established CKD may increase the extent of APOL1-induced injury after disease onset. Metabolic acidosis, a common manifestation of reduced glomerular filtration rate, has been shown to impair mitochondrial quality control and autophagy in renal tubular cells, potentially leading to mitochondrial dysfunction and oxidative stress. In vitro studies have revealed that metabolic acidosis may compromise mitochondrial autophagy and heighten tubular injury, thus providing a pathway through which acid retention in CKD might worsen APOL1-related metabolic stress [47]. In addition, the pH-dependent membrane-insertion characteristics of APOL1 channels suggest that local acidification in renal microenvironments might contribute to the initiation of harmful channel activity, thereby exacerbating ionic imbalance and cellular injury. Similarly, changes in potassium regulation could be another factor that alters the level of toxicity caused by APOL1. Variants of APOL1 that increase disease risk are known to cause cells to release potassium, and low intracellular potassium levels are among the main signals that activate the inflammasome and promote inflammatory cell death. Since low whole-body potassium levels contribute to high blood pressure and worsening of CKD, changes in potassium balance could further lower the threshold for APOL1 to cause damage. While these ideas have not been fully tested, they point to potentially adjustable metabolic factors that might affect disease progression in people with APOL1 high-risk genotypes.

An important unresolved question is how interferon-driven APOL1 induction and mitochondrial dysfunction interact to define high-risk cellular states. A plausible model is that interferon signaling drives APOL1 expression above a pathogenic threshold while simultaneously altering the cell’s metabolic and proteostatic state, thereby lowering tolerance to APOL1-mediated stress [18,28]. In this context, mitochondrial dysfunction may act both as an early consequence of APOL1 risk variant expression and as a determinant of cellular vulnerability [9,20,30]. Once mitochondrial membrane potential, ATP production, and redox balance decline beyond a critical point, podocytes may lose the capacity to compensate for APOL1-induced ion flux disturbances, proteotoxic stress, and inflammatory signaling [9,30,31]. This creates a tipping point at which initially adaptive stress responses become maladaptive, leading to feed-forward injury, loss of podocyte integrity, and irreversible cellular damage [28,31].

### 2.5. Autophagy, ER Stress, and Proteostasis Disruption

Cellular proteostasis disruption is now considered a major downstream consequence of APOL1 risk variant expression, with mitochondrial dysfunction, membrane damage, and inflammation contributing to this disruption. Autophagy, ER stress pathway activation, and the accumulation of misfolded/aggregated proteins lead to the buildup of damaged proteins within podocytes, triggering a “proteotoxic stress” response.

Multiple experimental studies using podocyte cell lines and transgenic mouse models expressing APOL1 risk variants have demonstrated impaired autophagic flux, accumulation of damaged mitochondria, and defective mitophagy [8,9,20]. Autophagy, a major homeostatic mechanism for maintaining healthy cellular systems, clears damaged organelles, specifically mitochondria, and removes improperly folded proteins from cells. In podocytes and renal epithelial cells containing the APOL1 G1 and G2 variants, there has been an increase in autophagosome accumulation, while the completion of autophagy’s degradation of damaged organelles and improperly folded proteins is compromised [20,37,46,48]. This is believed not to be related to an increase in autophagic activation but rather to an impediment to the flow of material through the autophagic process. The impaired flow of material through the autophagic process is mechanistically due to ATP depletion and impaired mitochondrial function, as autophagy is an energy-intensive process that relies on the cell’s bioenergetic components [46,49,50,51]. If damaged mitochondria are not adequately cleared from the cell, this will increase ROS production, thereby increasing metabolic stress and creating a cycle of gradually increasing cellular injury.

In parallel, APOL1 risk variants robustly induce ER stress and activate the unfolded protein response (UPR). In response to the accumulation of misfolded proteins in the ER lumen due to the expression of G1 or G2 variants, typical UPR signaling via PERK, IRE1α, and ATF6 is triggered [46,48,52]. Inducing the UPR transiently can have adaptive effects. However, with prolonged ER stress induced by APOL1 expression, the adaptive/maladaptive balance shifts in the final stages, leading to maladaptive effects (translation inhibition, deregulated lipid metabolism, and activation of pro-apoptotic signaling). Importantly, ER stress in APOL1-associated kidney disease is closely linked to impaired proteostasis and altered protein folding dynamics of the APOL1 protein itself, particularly involving the structurally destabilized C-terminal domain of the risk variants [46,48,52,53].

Proteotoxic stress resulting from the combined failure of autophagy and persistent ER stress is a major driver of cellular collapse. Biochemical and cell-based studies have demonstrated that APOL1 risk variants exhibit altered proteostasis, including increased susceptibility to misfolding, impaired degradation, and accumulation under stressful conditions [26,54]. These defects overwhelm cellular quality-control systems, leading to activation of stress kinases, disruption of cytoskeletal integrity, and sensitization to inflammatory and lytic cell-death pathways. Notably, proteostasis failure also intersects with APOL1 channel activity and mitochondrial injury, as ionic imbalance, ROS generation, and ATP depletion further compromise protein folding and degradation capacity [49,54].

Owing to their status as terminally differentiated cells with limited regenerative capacity, podocytes are at heightened risk due to the coalescence of autophagy, ER stress, and abnormal regulation of protein homeostasis (proteostasis). As such, podocytes rely significantly on functional proteostasis networks to sustain the integrity of their cytoskeletal infrastructure and regulate the turnover of slit diaphragm proteins. Disruption of either the cytoskeleton or associated networks results in cytoskeletal disintegration, effacement of foot processes on podocytes, detachment of podocytes from the glomerular basement membrane, and ultimately, podocyte death, hallmarks of CKD progression [55,56]. Evidence from the use of experimental animal models expressing high-risk variants of APOL1 within podocytes indicates that abnormal protein regulation occurs before both the loss of podocytes and the manifestation of glomerulosclerosis, thereby providing further evidence for an early or proximal effect of abnormal protein regulation in mediating podocyte injury, rather than being a consequence of podocyte injury [6].

### 2.6. Inflammatory and Innate Immune Signaling Pathways

The inflammatory/innate immune signaling pathways are important for the discovery and amplification of APOL1-mediated kidney injury. Experimental studies have shown that interferon stimulation markedly increases APOL1 expression through JAK–STAT signaling, thereby linking environmental inflammatory triggers to APOL1-mediated podocyte injury [7,18]. The *APOL1* gene is interferon-responsive, and its expression is linked to the activation of the innate immune system. Thus, immune activation is not merely a byproduct of APOL1-related kidney disease but rather directly contributes to its onset, severity, and progression.

Transcriptomic studies of human kidneys, both at the tissue and individual cell levels (using bulk and single-cell RNA sequencing), have shown that ISG expression is consistently clustered with APOL1 expression in podocytes, endothelial cells, and tubular epithelial cells during inflammation [34,35]. This known coupling between APOL1 and ISGs is likely the basis for the strong correlation between the APOL1 high-risk genotype and kidney diseases that exist in interferon-rich clinical settings.

Interferon signaling not only increases the total amount of APOL1 protein in the cell, but also alters the cellular context, making cells progressively more vulnerable to APOL1 toxicity. The activity of interferons leads to numerous alterations in cellular metabolism and proteostasis, among other things, by reducing the global rate of protein translation, activating oxidative stress response pathways, and modulating autophagy. In this setting, APOL1 risk variants are more likely to exceed the pathogenic expression threshold and engage downstream toxic mechanisms, reinforcing the “second-hit” paradigm [7,57,58].

In addition to its role in interferon signaling, APOL1 risk variants are associated with inflammatory pathways that require activation of nuclear factor kappa-light-chain-enhancer of activated B cells (NF-κB) and the inflammasome, leading to increased injury. As NF-κB is a major modulator of the innate immune response, multiple pathways activate NF-κB, all of which are relevant to APOL1-associated diseases. Viral infections, cytokine release, oxidative stress, and cell injury all lead to NF-κB activation. The results from the studies to date indicate that the expression of APOL1 risk variant(s) can increase NF-κB pathway activity, either directly by stimulating it or indirectly through increased oxidative stress, mitochondrial damage, ROS production, and ER stress [28,59,60]. Activation of NF-κB leads to the production of several cytokines and chemokines, as well as other response genes, creating an environment within the glomerulus that induces injury by promoting inflammation.

The NLR family pyrin domain-containing 3 (NLRP3)-related Inflammasome pathways are another direct connection between APOL1 toxicity and the innate immune system. Potassium efflux induced by APOL1 channel activity promotes inflammasome activation. In podocytes, as in all types of renal cells, loss of potassium via APOL1 has been shown to drive inflammasome signaling and activate IL-1β and IL-18 maturation via caspase-1, initiating lytic inflammatory cell death programs [10,44]. By introducing these pathways into tissue injury and inflammatory signaling in the kidney, APOL1 exacerbates the problem.

A defining feature of APOL1-associated kidney disease is the presence of feed-forward inflammatory loops that amplify injury once they are initiated. Interferon signaling induces APOL1 expression. APOL1 risk variants subsequently activate multiple downstream stress pathways. These stress signals activate NF-κB and inflammasome pathways, leading to cytokine and interferon production, which further enhances APOL1 expression and inflammatory signaling [7,9,10]. This self-reinforcing cycle helps explain why relatively transient inflammatory insults can precipitate sustained kidney injury in genetically predisposed individuals. Such feed-forward loops also provide a mechanistic explanation for the progressive nature of APOL1-associated diseases. Even if the initiating inflammatory trigger resolves, downstream stress pathways and maladaptive immune activation may persist, driving podocyte loss, glomerulosclerosis, and CKD progression [3,28].

The intimate relationship between innate immune signaling and altered APOL1 levels is most clearly demonstrated in viral and autoimmune diseases. HIVAN, which occurs almost exclusively in patients harboring one or both of the APOL1 high-risk genotypes and exposed to long-term chronic infection with HIV, as well as to the continued introduction of interferons, represents the most penetrant clinical manifestation of kidney disease associated with the APOL1 locus [1,4]. A more recent example is collapsing glomerulopathy associated with SARS-CoV-2 (COVID-19) infection, in which strong activation of the innate immune system, as well as continued interferon signaling, can unmask podocyte injury associated with APOL1 [39,61,62,63].

Autoimmune diseases and therapeutic interferon exposure similarly highlight the pathogenic synergy between immune activation and the APOL1 genotype. Cases of collapsing glomerulopathy and rapidly progressive kidney disease have been reported in individuals with APOL1 high-risk genotypes following interferon therapy or in the setting of systemic autoimmune inflammation, reinforcing the concept that immune-mediated stimuli can drive APOL1 expression beyond the pathogenic threshold [25,28,61].

**Figure 1:**
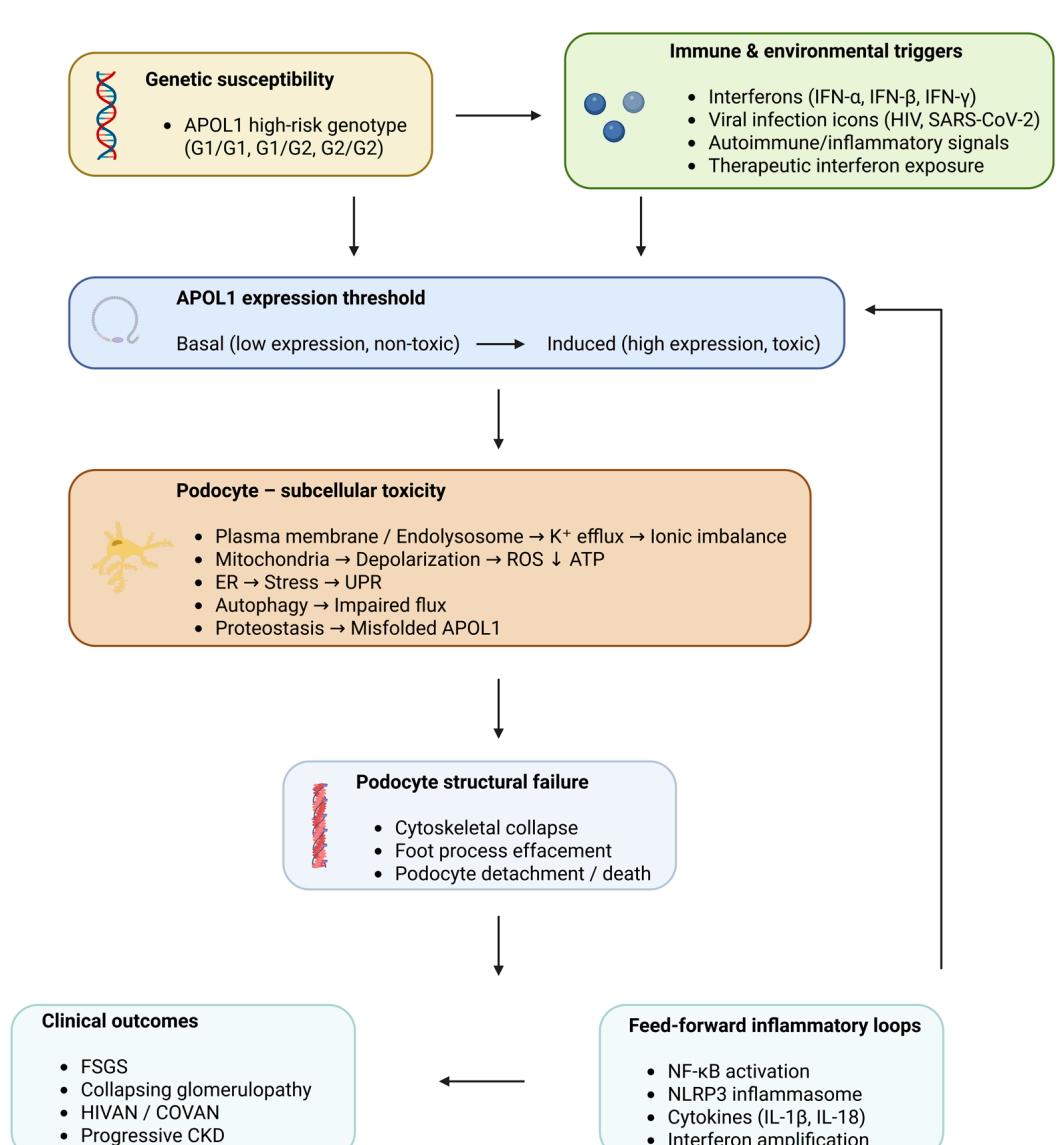
The APOL1 integrated pathogenic model of kidney disease. The integrated pathogenic model includes the contribution of high-risk APOL1 genotypes (G1 and G2) to kidney disease, but they are not pathogenic in the absence of a second trigger. An example of a second trigger is the activation of the innate immune system, specifically the signaling cascade through interferons, which occurs with viral infection, autoimmune inflammation, or the therapeutic use of interferons. This immune system activation would increase APOL1 levels above those considered pathogenic. The pathogenic *APOL1* gene causes a toxic gain-of-function in podocytes. However, there are also many aspects of toxic gain-of-function in podocytes beyond the pathogenic *APOL1* gene. Toxic pathways converge to destabilize the podocyte actin cytoskeleton, leading to effacement of podocyte foot processes, detachment from the glomerulus, and loss of podocytes. The cation channel-mediated efflux of potassium ions, depolarization of mitochondrial membranes, ROS production, loss of oxidative phosphorylation, increased ER stress, impaired flow of autophagy, and inability to maintain proteostasis are all examples of toxic pathways. Activation of NF-κB and inflammasome pathways creates feed-forward inflammatory and interferon signaling loops that sustain APOL1 expression and injury even after the initiating trigger resolves. Over time, the progressive loss of podocytes leads to FSGS, collapsing glomerulopathy, and ultimately to CKD.

Collectively, these interconnected pathways converge on podocytes to drive cytoskeletal collapse, podocyte loss, and progressive glomerular injury, providing a mechanistic basis for the heterogeneity and progression of APOL1-associated kidney disease (Figure 1).

### 2.7. Experimental Models of APOL1-Associated Kidney Disease

Experimental models have played a central role in elucidating the molecular mechanisms underlying APOL1-mediated kidney injury. Because rodents do not naturally express APOL1, transgenic approaches have been required to investigate the pathogenic effects of human APOL1 risk variants in vivo. Independent mouse models have been created in which human APOL1 alleles are expressed either specifically in podocytes or under inducible promoters. These models have given direct experimental proof that APOL1 risk variants lead to a toxic gain-of-function in renal cells [6,10,38]. Transgenic mice expressing podocyte-specific APOL1 G1 or G2 variants develop proteinuria, podocyte foot process effacement, and progressive glomerulosclerosis, thereby closely mimicking the major pathologic changes in human APOL1-related kidney disease. It is notable that, in the same models, expression of the normal APOL1 allele does not lead to similar renal damage, thereby providing robust experimental evidence for a toxic mechanism specific to the variant [6]. Experimental models further demonstrate that APOL1 toxicity is strongly dose dependent. Systems with regulated APOL1 expression show that cells can cope with low levels of APOL1 expression most of the time, whereas high levels of APOL1 expression lead to mitochondrial dysfunction, cytoskeletal disruption, and activation of inflammatory signaling pathways [9,10,40]. The data are consistent with a model in which APOL1 expression must exceed a threshold for pathogenic effects to be observed. In addition to transgenic mice, a series of in vitro systems, such as cultured human podocytes, renal epithelial cell lines, and induced pluripotent stem cell-derived kidney organoids [9,39], have yielded additional mechanistic data. These experimental systems confirm that APOL1 risk variants disrupt multiple cellular stress pathways in renal cells [8,39].

## 3. Future Perspectives: Toward Precision Medicine in APOL1-Associated Kidney Disease

There have been significant advances over the last 10 years that have changed the way we view APOL1-associated kidney disease and the collection of clinicopathologic diagnoses. Rather than viewing them as a collection of clinicopathological diagnoses, we now consider them examples of genetically and molecularly defined diseases. As we gain more mechanistic insight into APOL1-associated kidney disease, the APOL1 field is now ready to shift from descriptive associations to precision-based risk stratification and, ultimately, to the development of therapies targeted at specific pathways. Several key areas will define future progress in this field.

A major implication of APOL1 biology is the need to redefine the disease classification. Currently available traditional diagnostic approaches and identification systems for kidney diseases, such as FSGS, HIVAN, collapsing glomerulopathy, and hypertension-related CKD, do not adequately capture the molecular mechanisms underlying abnormal kidney function in individuals carrying the APOL1 gene with an increased risk of kidney disease. Emerging data suggest that APOL1-related kidney disease is now recognized as a new class of kidney disease that can be defined as an endotype driven by specific genetic risk factors and molecular changes caused by immune activation (for example, increased interferon levels) [28,64]. In the future, new classification systems will likely consider both the genotype and inflammatory context, as well as molecular signatures, treatment.

The second significant challenge is to improve risk stratification beyond genotype alone. Even though carrying an APOL1 high-risk genotype significantly increases the chances of kidney disease, incomplete penetrance is still the major characteristic, whereby the majority of individuals who have the genetic predisposition do not develop clinically overt disease [3]. It is believed that several factors explain this incomplete penetrance. Firstly, APOL1 levels are normally kept under tight control and are usually low in unstimulated cells, so that they rise only upon inflammatory stimuli, e.g., viral infection, immune system activation, or interferon exposure, becoming pathogenic [3,28]. Secondly, genetic and epigenetic regulators could affect the APOL1 gene transcription and the ways in which cells react to stress [19,27]. Thirdly, environmental and clinical factors, including metabolic stress, coexisting kidney injury, and varying levels of immune activation, may, in conjunction with the genotype, contribute to the pathogenesis of the disease [14,28]. These uncertainties have important implications for genetic counseling and screening strategies. The presence of a high-risk APOL1 genotype should therefore not be interpreted as deterministic for kidney disease but rather as a susceptibility factor whose clinical impact depends on additional biological and environmental contexts. Consequently, genetic testing is most informative when integrated with clinical risk assessment, family history, and longitudinal monitoring of kidney function. Current guidelines, therefore, emphasize cautious implementation of APOL1 screening, particularly in transplantation and research settings, while avoiding stigmatization or overinterpretation of genetic risk in asymptomatic individuals [14,29].

This emphasizes the importance of identifying the biological factors that influence when and in whom a disease will show signs. By utilizing multicenter longitudinal datasets integrating transcriptomic, proteomic, and metabolomic information on an individual-cell basis and modelling these factors over time, researchers have the opportunity to better define the early molecular changes associated with the activation of the *APOL1* gene and cellular stresses likely to occur in the kidney before irreversible podocyte death [34,35,65]. In addition, such research designs may also enable scientists to develop biomarkers that, in the future, might inform a person’s risk of kidney disease by measuring APOL1 gene function, interferon exposure, and mitochondrial dysfunction. This way, patients at the highest risk of disease progression and those who remain stable can be identified clinically. Indeed, several potential biomarker types have surfaced from recent mechanistic and multi-omics investigations. To begin with, interferon-stimulated gene (ISG) patterns in plasma from the kidney or immune cells may serve as markers of the initial inflammatory activation that produces APOL1 and could expose individuals with active pathogenic second hits [34,35]. Secondly, indicators of podocyte damage, for instance, urinary proteins released by podocytes like nephrin fragments and podocin, may hint at glomerular barrier stress before significant podocyte loss occurs [55,56]. Third, mitochondrial stress markers, including circulating or urinary mitochondrial DNA fragments and metabolic signatures reflecting impaired oxidative phosphorylation, have been proposed as indicators of APOL1-associated metabolic injury [9,30]. Proteomic research has further revealed inflammatory and metabolic protein signals in the bloodstream, which correlate with APOL1 risk variants and the progression of kidney disease [42]. While these biomarkers are still under research, they offer a glimpse into early or possibly reversible phases of APOL1-induced renal damage and could be used to direct genotype-based treatment strategies.

The emergence of APOL1-targeted therapies further emphasizes the importance of timing and patient selection. A number of therapeutic approaches are currently in clinical trials that directly target APOL1 to mitigate its toxic effects. Some of these therapies include pharmacologic inhibition of APOL1 channel activity and gene-silencing methods [66]. Pharmacologic inhibition of APOL1 channel activity is one of the leading approaches being explored. The small-molecule inhibitor of APOL1 channel activity, inaxaplin (VX-147), has rapidly progressed through a phase 2 clinical trial of APOL1-mediated kidney disease, where the drug led to significant reductions in proteinuria [67], and this strategy is currently being further evaluated in the ongoing AMPLITUDE phase 2/3 trial (NCT05312879) [68]. At the same time, gene-silencing methods using antisense oligonucleotides and RNA interference are being developed to reduce APOL1 expression. For example, the antisense oligonucleotide AZD2373 (opemalirsen) is currently being investigated in the APPRECIATE clinical trial (NCT06824987) [69]. These strategies have demonstrated promising efficacy in preclinical models and have reached early clinical development phases [32,70]. Nevertheless, several issues remain unresolved before deciding on the long-term safety of a constantly suppressed APOL1. For example, the effect on innate immune defense against trypanosomal infection, the possibility of off-target gene silencing, how long the therapeutic response would last, and the practical aspects of carrying out the treatments on a large scale due to cost and access limitations. Altogether, these therapies demonstrate that understanding the biology of APOL1 enables genotype-based treatment. One big unknown factor about these treatment options is whether they will work better as preventive measures in people who are genetically high-risk or as treatments that can alter the progression of the disease after APOL1-linked kidney injury has occurred. Currently, APOL1-targeted drugs are primarily viewed as treatments for active, already occurring disease rather than preventative measures. This perspective stems from the fact that the main clinical research so far, such as the phase 2 trial of inaxaplin, has focused on patients with clearly identifiable APOL1-related kidney disease and proteinuria, thus proving that using drugs to block APOL1 can mitigate the injury process in the kidneys of patients who have already been affected. From a mechanistic point of view, this strategy is also in line with findings showing that APOL1 risk variants lead to the activation of long-lasting cellular stress signals, such as disrupted ion homeostasis, mitochondrial damage, and enhanced inflammatory pathways, which might be amenable to therapy even after the disease has started [6,9,37]. Meanwhile, using it preventively is still a very real theoretical option. Since it seems that APOL1 toxicity relies on inducible expression, crossing pathogenic thresholds, and inflammatory “second hits,” the early targeting of APOL1 expression or function may, at least in theory, prevent the injury from occurring before podocytes are permanently lost [5,18,28]. This preventive strategy might be especially suitable for genetically high-risk individuals who are, for example, exposed to very strong inflammatory triggers such as viral infection, interferon-rich autoimmune disease, or other highly activated immune responses [18,28]. However, to date, no clinical trial has demonstrated that APOL1-targeted therapy can prevent disease development in asymptomatic high-risk individuals, and the long-term safety, optimal timing, and risk-benefit ratio of such an approach remain unknown [14,29]. On balance, current data are in favor of APOL1-targeted therapies being primarily disease-modifying treatments for active disease, whereas preventive use is still mechanistically plausible but unproven. This separation in uses further underscores the urgency of discovering biomarkers that can pinpoint the early reversible phases of APOL1-mediated damage and help determine when intervention is most likely to yield benefits [34,40].

One major area of uncertainty regarding these treatment options is whether they would be best used as preventative therapies or as treatments to slow down disease progression once a patient has already developed CKD. As podocyte loss cannot be reversed, future studies are required to identify specific stages of kidney disease and/or biomarkers that indicate active APOL1-mediated damage, thereby determining when to administer these treatments. In parallel, combination strategies targeting both APOL1 expression and downstream inflammatory or metabolic stress pathways may be necessary to fully suppress disease-driving feed-forward loops [28]. The rationale for combination therapy arises from the network structure of APOL1-mediated injury. Induction of APOL1 acts as the primary driver. However, when expressed at harmful levels, risk variants will trigger multiple downstream stress pathways simultaneously, such as potassium efflux, mitochondrial dysfunction, ER stress, proteostasis failure, and NF-κB/inflammasome signaling, which, in addition to amplifying each other, can also lead to sustained injury. For instance, APOL1 induces ionic imbalance, supporting inflammasome activation, while mitochondrial dysfunction leads to oxidative stress and ATP depletion, which exacerbate proteostatic stress and inflammatory signaling. Then, interferon signaling further increases APOL1 expression, thereby forming a self-sustaining cycle. Such a mechanistic setup suggests that simply shutting down APOL1 expression may not be sufficient if downstream inflammatory or metabolic pathways have already been activated. Thus, treatments that target both APOL1 and the main downstream stress pathways may be necessary to fully break the disease, thereby promoting feed-forward loops [9,18,28,31,32].

Another key direction is integrating APOL1 biology into the broader field of precision nephrology. APOL1-associated diseases illustrate how genetic variation related to ancestry interacts with environmental factors, such as the immune system, to increase disease risk. With the expansion of precision medicine, ethical aspects such as equitable access to genetic testing, avoiding genetic determinism, and implementing genotype-guided therapies in ways that consider cultural context must be carefully considered [71,72]. Interventions targeting APOL1 should not be considered as having specific ancestry (e.g., African ancestry), but rather as therapies aimed at all individuals with similar molecular mechanisms and vulnerabilities to kidney disease.

The study of APOL1 highlights a broader scheme for understanding the role of gene–environment interactions in the pathogenesis of kidney malfunction. The concept of a second hit, which refers to the exposure of genetic susceptibilities to additional stressors (e.g., infection and immune stimuli), has implications that extend beyond APOL1 to other forms of CKD with variable penetrance. Insights gained from APOL1-associated diseases are therefore likely to inform strategies for dissecting heterogeneity, identifying modifiable triggers, and developing targeted therapies across nephrology [15,64].

## 4. Conclusions

Our understanding of APOL1-associated kidney disease at the molecular level has greatly improved over the last ten years. Human genetics, in vitro experiments, and animal studies using genetically modified lines have collectively confirmed that APOL1 risk variants induce a toxic gain-of-function in kidney cells, particularly in podocytes. APOL1 risk variants induce cellular injury through multiple interconnected molecular pathways. The pathways include ion transport dysregulation, mitochondrial dysfunction, reduced autophagy, proteostatic stress, and activation of innate immune signaling networks. These results underscore the importance of integrating experimental and clinical evidence in research on APOL1-associated kidney disease. While clinical studies have defined the spectrum of APOL1-associated nephropathies, experimental models have been essential for elucidating the mechanisms by which APOL1 risk variants drive cellular injury and disease progression. Experimental research across various experimental systems demonstrates a mechanism in which podocytes are the primary effectors of APOL1-mediated injury. Podocytes depend on the structural integrity of their cytoskeletons and the normal function of their mitochondria for both energy and homeostasis. APOL1 risk alleles disrupt multiple cellular homeostatic mechanisms essential for podocyte survival. Recent multi-omics and single-cell studies further support the concept that APOL1-associated kidney disease involves coordinated injury across multiple renal cell types rather than a strictly podocyte-autonomous process. Podocytes are the main kidney cells known to be affected in APOL1-associated injuries, but we now have substantial evidence that cells lining the blood vessels and kidney tubules may also be involved. Interferon, inducible APOL1 expression in glomerular endothelial cells has been correlated with endothelial stress, microvascular dysfunction, and disrupted podocyte-endothelial interactions. These findings suggest that endothelial injury could not only worsen glomerular damage but also determine the severity of the disease. Both inflammatory and ischemic conditions can convert tubular epithelial cells, typically low in APOL1 expression, into cells with elevated APOL1 expression. Apart from this, malfunctioning mitochondria or impaired healing processes in these cells could lead to the development of tubulointerstitial fibrosis and the worsening of long-term kidney diseases. All this data supports the idea of a complex disease model involving multiple cell types. Podocytes are the main cells that are damaged. However, endothelial and tubular cells also play major roles in disease progression and the development of chronic conditions. In some inflammatory scenarios, for example, large interferon release or viral infection, these additional cell types may not only be modulators, but also direct contributors to disease initiation.

A defining and unifying feature of APOL1 biology is its context dependence. Basal repression of APOL1 expression, coupled with potent inducibility by interferons and other inflammatory stimuli, creates a narrow pathogenic window in which risk variants exert toxicity. This regulatory architecture provides a compelling biological basis for the second-hit paradigm and explains the incomplete penetrance, phenotypic heterogeneity, and strong association with viral infection, autoimmune disease, and interferon exposure observed clinically. This framework illustrates how transient immune activation is an initiating event in the cascade leading to long-term kidney damage in patients with genetic predisposition.

From a translational perspective, APOL1-associated kidney disease represents a compelling model of precision nephrology, in which genetic risk stratification can be directly linked to mechanism-based therapies targeting APOL1 expression or channel activity. At the same time, these advances highlight the need to develop improved methods for molecular stratification of patients based on early identification of active APOL1-related damage (i.e., markers) and consideration of the timing of therapeutic intervention to prevent irreversible podocyte destruction.

Continued efforts to translate mechanistic findings into a clinically usable approach will not only benefit individuals at risk based on APOL1 genotype, but also advance the identification and characterization of unique disease presentations and gene/environmental interactions in patients with CKD.

## Figures and Tables

**Table 1:** Clinical phenotypes and triggers associated with APOL1 high-risk genotypes.

Clinical Phenotype	Strength of APOL1 Association	Key Triggers	Dominant Pathology	References
FSGS	Strong	Idiopathic or inflammatory contexts	Podocyte injury, segmental sclerosis	[1,2,3]
Collapsing glomerulopathy	Very strong	Viral infection, interferon exposure, autoimmune inflammation	Podocyte collapse, rapid progression	[2,4,17]
HIVAN	Strongest/most penetrant	Chronic HIV infection, sustained interferon signaling	Collapsing glomerulopathy, tubular microcysts	[2,15,18]
COVAN	Strong	SARS-CoV-2 infection, cytokine storm	Acute podocyte injury, collapse	[5,7,10]
Progressive CKD (often labeled hypertension-attributed)	Moderate-strong	Recurrent inflammatory or metabolic stress	Podocyte loss, interstitial fibrosis	[6,8]
IFN therapy-associated nephropathy	Strong (subset)	IFN-α/β/γ treatment	Collapsing or FSGS lesions	[19,20]

Abbreviations: APOL1, apolipoprotein L1; FSGS, focal segmental glomerulosclerosis; HIVAN, human immunodeficiency virus-associated nephropathy; SARS-CoV-2, severe acute respiratory syndrome Coronavirus 2; COVAN, COVID-19-associated nephropathy; CKD, chronic kidney disease; IFN, interferon.

**Table 2:** Cellular compartments involved in APOL1-associated kidney injury and major unresolved questions.

Renal Compartment	Evidence for APOL1 Involvement	Key Mechanisms	Strength of Evidence	Major Unresolved Questions	References
Podocytes	High APOL1 expression in podocytes; podocyte-specific expression of APOL1 risk variants in experimental models induces proteinuria and glomerulosclerosis	Ion channel activity, mitochondrial dysfunction, ER stress, impaired autophagy, and inflammasome activation	Strong (genetic, experimental, and clinical evidence)	Why do only a subset of high-risk individuals develop podocyte injury? What determines the pathogenic APOL1 expression threshold?	[6,7,34,35,36]
Glomerular endothelial cells	APOL1 expression is inducible by interferons; transcriptomic studies show endothelial stress and inflammatory activation in APOL1 high-risk contexts	Microvascular dysfunction, inflammatory signaling, and altered podocyte–endothelial crosstalk	Moderate (transcriptomic and experimental studies)	Is endothelial injury primary or secondary to podocyte damage? Does endothelial APOL1 expression independently drive disease progression?	[18,28,40]
Tubular epithelial cells	Low basal APOL1 expression but inducible during inflammatory or ischemic stress; tubular injury observed in APOL1-associated CKD and collapsing glomerulopathy	Metabolic stress, mitochondrial dysfunction, maladaptive repair, fibrosis	Emerging (experimental and observational evidence)	Does tubular APOL1 expression directly initiate injury, or mainly amplify chronic kidney damage?	[18,30,31,37]

Abbreviations: APOL1, Apolipoprotein L1; CKD, chronic kidney disease; ER, endoplasmic reticulum.

**Table 3:** Molecular mechanisms underlying APOL1 risk-variant toxicity and their cellular consequences.

Pathogenic Mechanism	Key Molecular Features	Affected Cellular Processes	Relevance to Podocytes	References
Gain-of-toxic-function.	Qualitative protein changes (G1/G2)	Membrane activity, altered localization	Explains disease without loss-of-function	[1,41]
Ion channel activity	pH-sensitive cation conductance, K^+^ efflux	Ionic imbalance, cell swelling	Triggers inflammasome activation	[27,42]
Mitochondrial dysfunction	Depolarization, ROS, ↓ OXPHOS	ATP depletion, metabolic stress	High energetic demand	[11,12]
Autophagy impairment	Blocked autophagic flux	Accumulation of damaged organelles	Promotes oxidative/proteotoxic stress	[13,16]
ER stress/UPR activation	PERK–IRE1α–ATF6 signaling	Translational arrest, apoptosis	Linked to APOL1 misfolding	[20,22]
Proteostasis failure	Misfolded protein accumulation	Cytoskeletal destabilization	Foot process effacement	[23,24]
Innate immune activation	NF-κB, NLRP3 inflammasome	Cytokine amplification	Feed-forward injury loops	[14,25,26]

Abbreviations: APOL1, apolipoprotein L1; G1, APOL1 G1 risk allele; G2, APOL1 G2 risk allele; K^+^, potassium ion; OXPHOS, oxidative phosphorylation; ROS, reactive oxygen species; ER, endoplasmic reticulum; UPR, unfolded protein response; PERK, protein kinase RNA-like endoplasmic reticulum kinase; IRE1α, inositol-requiring enzyme 1 alpha; ATF6, activating transcription factor 6; NF-κB, nuclear factor kappa-light-chain-enhancer of activated B cells; NLRP3, NLR family pyrin domain containing 3; ATP, adenosine triphosphate.

## Data Availability

No new data were created or analyzed in this study. Data sharing is not applicable to this article.

## Abbreviations

The following abbreviations are used in this manuscript:

APOL1	Apolipoprotein L1
ATP	Adenosine triphosphate
ATF6	Activating transcription factor 6
CKD	Chronic kidney disease
COVID-19	Coronavirus disease 2019
COVAN	COVID-19–associated nephropathy
DKD	Diabetic kidney disease
ER	Endoplasmic reticulum
FSGS	Focal segmental glomerulosclerosis
G1	APOL1 G1 risk allele
G2	APOL1 G2 risk allele
GWAS	Genome-wide association studies
HIV	Human immunodeficiency virus
HIVAN	Human immunodeficiency virus–associated nephropathy
IFN	Interferon
IFN-α	Interferon alpha
IFN-β	Interferon beta
IFN-γ	Interferon gamma
IRE1α	Inositol-requiring enzyme 1 alpha
ISG	Interferon-stimulated gene
JAK	Janus kinase
K^+^	Potassium ion
NAD^+^	Nicotinamide adenine dinucleotide
NADH	Reduced nicotinamide adenine dinucleotide
NF-κB	Nuclear factor kappa-light-chain-enhancer of activated B cells
NLRP3	NLR family pyrin domain containing 3
OXPHOS	Oxidative phosphorylation
PERK	Protein kinase RNA-like endoplasmic reticulum kinase
ROS	Reactive oxygen species
SARS-CoV-2	Severe acute respiratory syndrome coronavirus 2
STAT	Signal transducer and activator of transcription
UPR	Unfolded protein response
